# Implementation of a Hepatitis B Screening Program in Patients Receiving Systemic Anti-Cancer Therapy

**DOI:** 10.3390/curroncol32010020

**Published:** 2024-12-30

**Authors:** Jennifer Leigh, Ranjeeta Mallick, Stephanie Brule, Lisa Rambout, Jennifer Newton, Dominick Bossé, Curtis Cooper, Joanna Gotfrit

**Affiliations:** 1 Division of Medical Oncology, Department of Medicine, The Ottawa Hospital, Ottawa, ON K1H 8L6, Canada; jleigh@toh.ca (J.L.);; 2 Ottawa Hospital Research Institute, Ottawa, ON K1H 8L6, Canada; 3 The Ottawa Hospital Pharmacy Department, Ottawa, ON K1H 8L6, Canada; 4 The Ottawa Hospital, Ottawa, ON K1H 8L6, Canada; 5 Division of Infectious Diseases, Department of Medicine, The Ottawa Hospital, Ottawa, ON K1Y 4E9, Canada

**Keywords:** hepatitis B virus, hepatitis B viral reactivation, gastrointestinal cancer, colorectal cancer, screening

## Abstract

Cancer patients receiving non-endocrine therapies are at risk of hepatitis B virus (HBV) reactivation (HBVr). Guidelines recommend HBV screening prior to treatment. The Ottawa Hospital Cancer Center implemented a screening pilot for all patients receiving FOLFOX-based regimens between January and April 2023. We assessed the pilot from a quality improvement perspective. Charts were retrospectively reviewed, and patient and disease characteristics were collected. The primary endpoint was to identify the proportion of patients who underwent HBV screening prior to treatment start. Univariate analyses assessed the association between baseline characteristics and failure to screen. Quality metrics were also reviewed. There were 32/42 patients (76.2%) who completed screening, and 5 (11.9%) had a positive screen. The majority of eligible patients (59.5%) completed screening prior to the first treatment as intended. Four of five patients who tested positive were referred to Infectious Diseases. Of those, one received antivirals for chronic HBV. There were no treatment delays due to pending screening and no HBV reactivation. Receipt of prior systemic therapy was significantly associated with failure to screen (55 vs. 95%, OR 17.1 (95% CI 1.92–153), *p* = 0.011). The results of this pilot highlight the importance of building HBV screening into standardized treatment plans and engaging all team members to ensure high levels of screening. Prior systemic therapy receipt was associated with failure to screen, and thus, programs should include education on the necessity of screening as recommended by medical guidelines.

## 1. Introduction

Hepatitis B virus (HBV) infection is a vaccine-preventable viral infection that is transmitted through contact with infected bodily fluids and has the potential to cause life-threatening complications involving the liver [1]. Immunocompetent patients who are infected with HBV will clear the virus spontaneously over 95% of the time. The remainder will present either with acute symptomatic infection and/or develop chronic infection identified during screening or work-up of other diseases [1]. There is no cure for those who do end up developing an infection, but antiviral treatments are highly effective in controlling the infection, slowing progression, and improving survival [1,2]. The prevalence of HBV is much lower in Canada than in other parts of the world, such as East Asia and Africa, where the prevalence is the highest [1,2,3]. This is largely due to the nationwide vaccination program, as well as screening key individuals such as pregnant mothers and those at increased risk.

Patients with chronic or clinically resolved (past) HBV are at risk of HBV reactivation (HBVr), which is defined as a rise in HBV DNA compared to baseline or seroreversion from hepatitis B surface antigen (HBsAg) negative to HBsAg positive [4,5,6,7]. The clinical picture of HBVr can vary widely from asymptomatic hepatitis to potentially fulminant hepatic failure, which can be fatal [8,9]. Risk factors for HBVr include increased age, male sex, and receipt of immunosuppressive therapies. Identifying patients most at risk of HBVr is crucial given the potentially life-threatening complications and the fact that it is largely preventable with oral antiviral therapy.

Systemic therapies used to treat cancer often result in immune suppression, and thus, patients receiving these treatments are at an increased risk of HBVr [4,5,6,7,10,11]. The risk of reactivation depends on a number of factors, including cancer type, therapy received, and the patient’s virological and serological status. In patients with chronic HBV, the risk of reactivation is estimated to be 24–88% in those with hematologic malignancies who receive B-cell depleting agents, 53–72% in those with hematologic malignancies who receive therapies other than B cell depleting agents, and 4–68% in patients receiving therapy for solid tumors [4,5,6,7,10,11,12]. Based on this, it was recommended by the American Society of Clinical Oncology (ASCO) in 2020 that all patients anticipating (non-hormonal) systemic anti-cancer therapy should be tested for HBV prior to starting therapy or during the first cycle [11]. Patients with known or screen-positive chronic HBV infection (and some with past HBV Infection) should receive consideration for prophylactic antiviral therapy for the duration of their systemic treatment and up to 12 months after completion, and/or have enhanced laboratory monitoring, usually via referral to an HBV specialist [11].

Despite these universal HBV screening recommendations, the real-world uptake has been variable. A retrospective study by Hwang et al. examined screening rates for patients receiving chemotherapy at a large US cancer center between 2004–2011 [12]. Only 17% of all patients receiving chemotherapy were screened for HBV; however, this increased during the study period. That is likely in part due to the fact that the Centers for Disease Control and Prevention, National Comprehensive Cancer Network, American Association for the Study of Liver Diseases, and ASCO all released HBV screening recommendations during this period. These data are consistent with that seen in other studies, including one from a large United States hospital network, which found that 17.1% of patients were screened before chemotherapy, with the majority being patients with hematologic malignancies (55.6%) as opposed to solid tumor patients (8.3%) [13].

**Figure 1:**
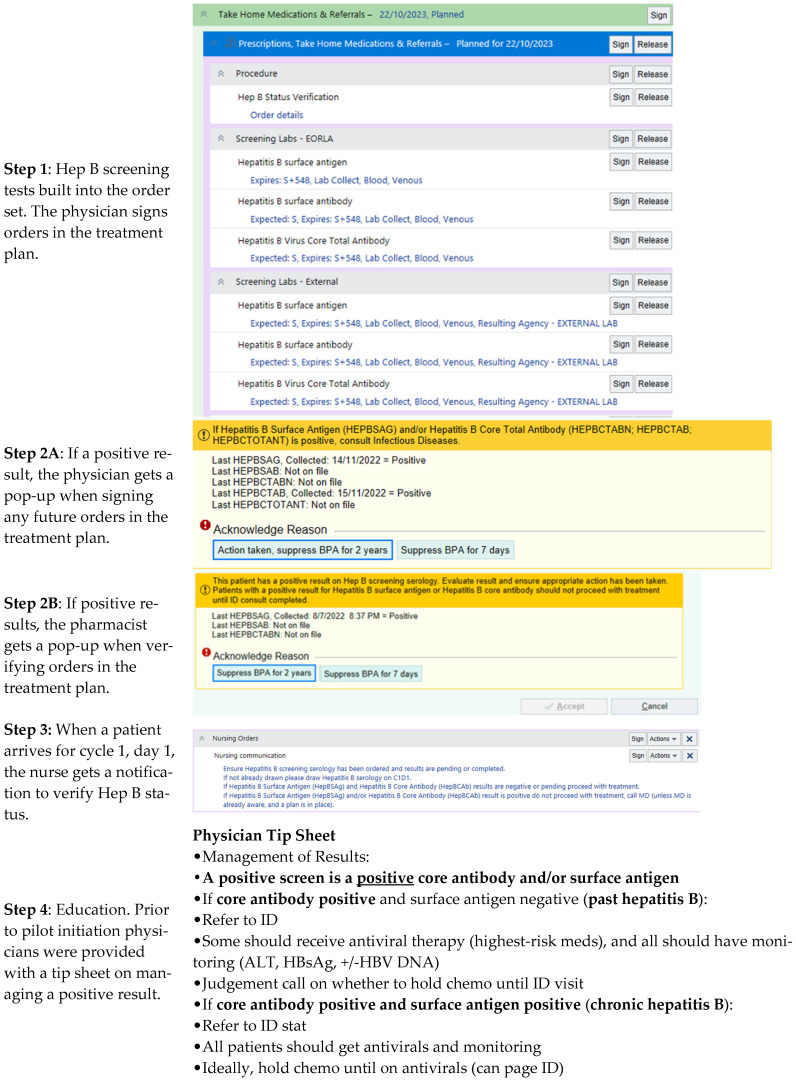
HBV Screening Process Overview. Outline of the HBV screening program, including steps taken, treatment plan orders, communications distributed, and best practice advisories received by various members of the healthcare team.

In 2023, The Ottawa Hospital Cancer Center implemented a three-month HBV screening pilot program for solid tumor patients. The screening program was developed by a multidisciplinary group including physicians, nurses, pharmacists, and managers, with consultation from Information Technology Services and Laboratory Services. The goal of the pilot program was to investigate the feasibility and efficacy of implementing a scalable HBV screening process at the initiation of chemotherapy in patients who were receiving a FOLFOX- (5-FU, leucovorin, oxaliplatin) based chemotherapy regimen. The screening lab tests (hepatitis B surface antigen, hepatitis B core antibody, hepatitis B surface antibody) were built into all included treatment plans, and both a pharmacy and nursing directive were implemented to ensure tests had been drawn prior to treatment start (or at the time of treatment initiation if not already done) and acted upon by the ordering physician if positive. At the time of chemotherapy verification, the pharmacist was directed to check for HBV screening results and, if positive, ensure the physician was aware and had taken the intended next steps. At the time of chemotherapy administration, the nurse was directed to check for HBV screening results and, if positive, to ensure the physician was aware and had acted upon the result prior to administering chemotherapy. If the HBV screening tests had not yet been drawn, they were drawn on the day of treatment in the chemotherapy unit by the nurse. If the results were pending or negative, the nurse proceeded with chemotherapy administration as per usual practice. A “best practice advisory” was also created to trigger a pop-up message in the electronic medical record if any positive screening results were detected. Prior to program implementation, educational sessions were provided to the physicians, nurses, and pharmacists, which included presentations and written handouts. A summary of this process and examples of the HBV notifications each healthcare team member received on the EMR can be seen in Figure 1.

To assess the success of the real-world implementation of this 3-month hepatitis B screening pilot program and to ascertain whether an institutional HBV screening policy may lead to screening in all eligible patients, we performed a retrospective chart review at the completion of the pilot program.

## 2. Materials and Methods

Ethics approval was obtained through the Ottawa Health Science Network Research Ethics Board. We performed a retrospective single-center chart review of the implementation of a Hepatitis B pilot program at The Ottawa Hospital, which included all patients with solid tumors who received a FOLFOX-containing regimen at The Ottawa Hospital or its satellite sites from 11 January 2023 to 11 April 2023. The Ottawa Hospital oncologists prescribe and oversee systemic therapy delivery both at The Ottawa Hospital Cancer Center and multiple satellite community sites at smaller hospitals. Patients have the option to complete their blood work at either the cancer center laboratory (EORLA) or externally, which includes community hospitals and commercial laboratories such as Lifelabs and Dynacare.

Patients included in the screening pilot program were those who received one of the following regimens between 11 January 2023 and April 2023: FOLFOX (adjuvant or palliative), FOLFOX + bevacizumab, FOLFOX + panitumumab, FOLFOX + nivolumab, FOLFOXIRI, and FOLFOXIRI + bevacizumab. The regimens chosen for inclusion in the pilot were arbitrarily chosen based on anticipated treatment volumes and expertise of the physicians involved in the pilot in order to conduct a small-scale trial of the screening program while avoiding widespread implications of unanticipated issues prior to complete rollout. Patients were excluded if they did not receive at least one cycle of the eligible regimens, if they received treatment outside of The Ottawa Hospital and its satellite sites, and if they were known to have either acute or chronic HBV prior to initial medical oncologist consultation.

Data on patient and disease characteristics collected at baseline included sex, age at diagnosis, age at treatment initiation, histopathologic diagnosis, stage at diagnosis and at treatment initiation, intent of therapy, timing of therapy, Eastern Cooperative Oncology Group (ECOG) performance status (PS), treatment start date, and whether prior systemic therapy was received. The ECOG PS, when not directly stated in the clinical notes, was inferred based on the assessment recorded at the time of the treatment decision. Data collected pertaining to the screening program included the date testing was ordered, date drawn, location drawn, number of times tested, whether testing occurred prior to treatment or in the chemotherapy unit, date of screening result, result of screening, infectious disease referral, antiviral prescription, and whether HBVr occurred. The primary endpoint was the proportion of patients who underwent HBV screening at the initiation of treatment. Secondary endpoints included the proportion of patients with a positive HBV screen, the proportion of patients who experienced a chemotherapy delay due to screening, and the proportion of patients who were started on antivirals for HBV.

Descriptive statistics were used to describe patient and disease characteristics. Categorical variables are reported as number (*n*) and proportion (%), and continuous variables are reported as the median and standard deviation (SD). Factors associated with HBV screening and HBV positivity were examined using univariate logistic regression models. Statistical significance was defined as *p* ≤ 0.05.

## 3. Results

### 3.1. Patient and Disease Characteristics

Patient and disease characteristics are described in Table 1. In total, there were 42 patients included in the pilot project. The median age was 64 years (range 42–82), the majority were male (*n* = 24, 57.1%), and the majority had an ECOG PS of 1 (*n* = 28, 66.7%). Most patients had a colorectal primary (*n* = 30, 71.4%), with other primaries including gastroesophageal (*n* = 9, 21.4%), appendix (*n* = 2, 4.76%), and pancreas (*n* = 1, 2.3%). Approximately two-thirds of the cohort were receiving palliative intent chemotherapy (*n* = 27, 64.3%). The predominant regimen received was mFOLFOX (*n* = 35, 83.3%). Other regimens received included mFOLFOX + bevacizumab (*n* = 3, 7.14%), mFOLFOX + nivolumab (*n* = 3, 7.14%), and FOLFOXIRI (*n* = 1, 2.38%). Slightly less than half of patients had received prior systemic therapy (*n* = 20, 47.6%).

### 3.2. Hepatitis B Virus Screening

Of the 42 patients included in the pilot, 32 underwent HBV screening (76.2%). Eight patients did not have screening labs ordered, and 2 patients had screening labs ordered that were never drawn. There were 25 patients who had screening completed as intended prior to starting systemic therapy (59.5%). Over a third of patients had their testing done in the chemotherapy unit on the day of treatment (*n* = 16, 38.1%). The median time to testing was 7.5 days (0–98 days), and the median time from testing to result was 3.0 days (1–6 days). There were 5 patients (11.9%) who had a positive HBV screen. One of these patients had chronic HBV, and the other four had clinically resolved (past) HBV. Of the screen-positive patients, 4 were referred to Infectious Diseases (80%), and only 1 of those referred was started on antiviral therapy (25%). This was the patient with chronic hepatitis B (HbsAg+). There were no patients who had a delay in their chemotherapy start due to pending or incomplete HBV screening, and no patients had HBVr during the study period. Further details on the results of the HBV screening pilot can be found in Table 2.

### 3.3. Factors Associated with Hepatitis B Screening and Positivity

Factors associated with completed HBV screening were examined using univariate analysis (Table 3). The only factor found to be significantly associated with screening completion was the lack of receipt of prior systemic therapy. Of the 20 patients who had received prior systemic therapy, 11 (55%) were screened for HBV. In comparison, there were 22 patients who had never received systemic therapy, and 21 (95.5%) of these patients were screened for HBV (OR 17.1 (95% CI 1.92–153), *p* = 0.011). The other factors examined included age, sex, site of primary disease, stage, intent of therapy, timing of therapy, and regimen received, none of which were significantly associated with screening completion. Potential factors associated with positive HBV screening were also examined using univariate analysis (Table 4), including sex, primary disease site, stage, intent of therapy, timing of therapy, and prior systemic therapy receipt, none of which were significantly associated with HBV positivity.

### 3.4. Location of Screening

Patients in the pilot program could be screened at either the academic cancer center lab (EORLA) or an external lab (Lifelabs, Dynacare, satellite hospitals). The differences in screening success based on laboratory use were examined. There was no significant difference in the proportion of successfully screened patients with screening tests ordered at EORLA (*n* = 23, 95.8%) versus external labs (*n* = 9, 90.0%). Time to screening result took, on average, 2 days longer when the testing was performed at EORLA (3.0 days vs. 1.0 day); however, this difference was not statistically significant (Table 5).

## 4. Discussion

The timely identification of chronic or clinically resolved (past) HBV in patients planning to start systemic anti-cancer therapy is an important step in preventing HBVr, which can be associated with significant morbidity and mortality. Our study explores the success of a single-center HBV screening pilot program, which included patients who were planning to start a FOLFOX-containing systemic therapy regimen. We found that the implementation of an HBV screening program resulted in a successful screening rate of approximately three-quarters of eligible patients, of which 15.6% of patients screened positive, a value higher than we expected based on population statistics [14]. There were no treatment delays due to HBV screening, likely due to the provider communications we added to the treatment plan orders, and no patients developed HBVr during the study period. Lastly, the only factor that was found to be statistically significantly associated with successful screening was a lack of prior systemic therapy.

### 4.1. Success of HBV Screening

In this pilot program, HBV screening tests were built into all the included chemotherapy treatment plans. To ensure screening was ordered, completed, and acted upon if positive, both a pharmacy and nursing directive to check the results before chemotherapy verification and administration were implemented (Figure 1). Despite this, almost a quarter of patients did not undergo screening, a group comprised largely of patients who had received prior systemic therapy. While the reasons for the lack of screening were not readily available in the electronic medical record, we postulate that physicians may have felt the risk of HBVr was low in these patients as they had previously tolerated systemic therapy. Additionally, it has been reported in prior literature that the risk of HBV reactivation in solid tumors is underestimated by medical oncologists, which may have also played a role [15,16]. Tolerance of prior systemic therapy should not necessarily negate HBV screening, however, as some therapies, such as anthracyclines, have been reported to confer a higher risk of HBV reactivation [4]. Organizations such as Cancer Care Ontario (CCO) recommend considering repeat screening when starting a new line of systemic therapy [10]. Additional reasons for the lack of screening include the fact that implementation of a new process takes time to be ingrained into routine practice, and this pilot only looked at the first few months of implementation.

Another potential reason for missed screening could have been low perceived benefit by the medical oncologist in those without clear risk factors, which is a previously described criticism of universal HBV screening [17]. While we did not explore traditional hepatitis B risk factors in this study as they were not available in the electronic medical record, prior evidence has suggested that a risk-adaptive approach to screening is inadequate. A study by Ramsey et al. found that 21% of patients with chronic HBV lacked traditional risk factors [18]. A separate study by Hwang et al. found that when they attempted a selective screening approach, most patients had at least one significant risk factor requiring HBV screening, making selective screening inefficient [19]. As such, universal screening is recommended by multiple oncology organizations, including ASCO and CCO [10,11].

Despite the fact that 23.8% of patients were not successfully screened, this pilot had much higher rates of screening than similar published screening programs. A single-center study by Lee et al. examined rates of HBV screening before and after implementation of a screening program and found that after screening program implementation, only 31% of patients were successfully screened [15]. This was compared to rates of left ventricular function (LVEF) testing for patients undergoing cardiotoxic chemotherapy, for which 100% of patients underwent LVEF screening [15]. The suspected reasons for this discrepancy were low provider buy-in and difficulty changing physician practices. On the contrary, our study suggests that the implementation of these screening programs can be successful. Key factors that may have led to this success are the comprehensive and detailed screening process, including the integration of the screening tests directly into the treatment plans in the chemo ordering system, making it easy and seamless for physicians to adopt the process into their practice, and a multidisciplinary approach to planning and implementation.

### 4.2. HBV Prevalence

It is estimated that approximately 2% of the Canadian population is infected with HBV [3,20]. Interestingly, the 11.9% rate of HBV positivity was much higher in our study than in the general population. This raises the question as to whether the incidence of clinically unrecognized HBV could be higher in the oncology population. There have been other retrospective studies that have also identified a higher prevalence of chronic or clinically resolved HBV in the oncology population. However, to our knowledge, our study is the first to examine this in a specifically Canadian population [18,21]. Additionally, there are previous cohort studies that suggest an association between HBV and gastrointestinal cancers, specifically liver, biliary tract, colon, and pancreatic [22]. Our cohort was enriched for gastrointestinal cancers. Importantly, our study did have low numbers, and thus, further research is required to better understand this relationship. Additionally, other important risk factors for HBV, such as place of birth, were not able to be assessed and can also impact these results.

### 4.3. Quality Metrics and Quality Improvement

The main focus of our initiative was to identify areas for improvement at a systems level in order to develop the program into an efficient, seamless, and safe process. It was our intention for patients to have their HBV screening tests drawn prior to the day of their first treatment in order to increase the chances of having results by the first day of treatment and to minimize lab work drawn in the chemotherapy suite. Over one-third of the cohort, however, ultimately had their testing drawn at the time of treatment in the chemotherapy suite. Ideally, these tests would be drawn days earlier, either at the time of initial consultation or the patients’ other pre-systemic therapy blood work, to reduce the potential for delays and the burden placed on nurses administering the systemic therapy. Based on the short testing-to-result turnaround time in our pilot (3 days), this may permit having more results available at the time of initial treatment. That said, guidelines do not suggest delaying treatment for pending results unless the patient is awaiting a bone marrow transplant or has unexplained hepatitis [10,11]. Interestingly, while both external private labs and the hospital-based lab (EORLA) had quick turnaround times, the time from testing to result was found to be shorter for the external labs on average. This is potentially because some labs may send their samples to Public Health Ontario for processing, whereas others may complete their testing in-house, with potentially different processes and timelines. An exploration of lab processing was beyond the scope of our analysis.

There was a small proportion of patients who had testing completed twice (9.52%) inadvertently. This was predominantly because testing was completed at an external lab but not yet visible in the electronic health record to the healthcare team at the chemotherapy suite, leading to it being redrawn according to the specified pilot process. Unfortunately, it is not possible for the cancer center healthcare team to see tests (in our EHR) that are pending at an external lab. Since the time of the initial pilot program, our center has endeavored to complete testing at the hospital lab EORLA whenever possible, which should help reduce the number of unnecessary duplicate tests.

### 4.4. Future Challenges and Directions

With the success of the HBV screening pilot program and ongoing refinement with Plan-Do-Study-Act (PDSA) cycles, the program will be implemented for all patients undergoing non-endocrine anti-cancer therapy at our tertiary care hospital. Challenges in the scale-up endeavor include developing sufficient IT resources to build the screening tests and directives into the electronic chemotherapy ordering system for all applicable regimens and obtaining the laboratory resources necessary to efficiently run larger quantities of HBV screening tests in a timely manner. We plan to re-evaluate the success of the screening program following expansion on a larger scale. Additionally, it would be important to explore the impact that the HBV screening program has on the workload of physicians, pharmacists, and nurses in order to ensure that the process remains efficient and manageable when scaled up.

### 4.5. Limitations

Limitations of this study include its retrospective single-center nature. Additionally, it has a small sample size and was limited to patients with a gastrointestinal solid tumor based on the systemic therapy regimens that were included in the pilot program. Scalability was also not demonstrated in this small pilot, and thus, it will be important to continue evaluating this screening program as it is expanded. Additionally, while there were no patients in this study who experienced HBVr, the overall number of HBV-positive patients was low, and thus, it will be important to continue to monitor the occurrence of this upon expansion.

## 5. Conclusions

Oncology patients with chronic or clinically resolved HBV infection receiving non-hormonal anti-cancer therapies are at increased risk of HBV reactivation. Therefore, it is important to identify these patients early so that this risk can be mitigated with the use of antivirals and/or enhanced monitoring. Our study demonstrates the success of a single-center HBV screening pilot program and may serve as a building block for other centers and for future screening program development. Additionally, it highlights the value of broad interprofessional stakeholder collaboration in ensuring the success of screening programs, as well as starting small and scaling up. It also highlights areas for quality improvement, including the need to improve screening rates for those with prior systemic therapy exposure. Lastly, the rates of HBV infection may be higher in the oncology population than previously expected, further supporting the implementation of an HBV screening program for solid tumor patients.

## Figures and Tables

**Table 1:** Patient and Disease Characteristics. Data is represented as the total number of patients and percentage of cohort unless otherwise specified.

Characteristic	Description	Total
Age at diagnosis	Median (range)	64 (42–82)
Sex—no. (%)	Female	18 (42.9)
	Male	24 (57.1)
Primary—no. (%)	Appendix	2 (4.8)
	Colorectal	30 (71.4)
	Gastroesophageal	9 (21.4)
	Pancreas	1 (2.4)
ECOG Performance Status—no. (%)	0	11 (26.2)
	1	28 (66.7)
	2	3 (7.1)
Intent of Therapy—no. (%)	Curative	15 (35.7)
	Palliative	27 (64.3)
Timing of therapy—no. (%)	Neoadjuvant-Adjuvant	15 (35.7)
	First-Second Line	27 (64.3)
Stage at start of FOLFOX regimen—no. (%)	II	2 (4.8)
	III	12 (28.6)
	IV	28 (66.7)
Prior systemic therapy received—no. (%)	No	22 (52.4)
	Yes	20 (47.6)
Regimen received—no. (%)	mFOLFOX	35 (83.3)
	mFOLFOX + BEV	3 (7.1)
	mFOLFOX + NIV	3 (7.1)
	FOLFOXIRImFOLFOX + PMAB FOLFOXIRI + BEV	1 (2.4) 0 (0) 0 (0)

Abbreviations: ECOG: Eastern Cooperative Oncology Group, BEV: Bevacizumab, NIV: Nivolumab, PMAB: panitumumab.

**Table 2:** Hepatitis B Screening Program Quality Metrics. Data is represented as the total number of patients and percentage of those patients unless otherwise specified.

Quality Metric	Description	Total
Time to testing	Median Days (range)	7.5 (0–98)
Time to result	Median Days (range)	3.0 (1–6)
Location tested—no. (%)	EORLA	24 (57.1)
	External Lab	10 (23.8)
Hep B screening completed—no. (%)	No	10 (23.8)
	Yes	32 (76.2)
Hep B Screening completed prior to treatment start—no. (%)	No	17 (40.5)
	Yes	25 (59.5)
Hep B Screening drawn more than once—no. (%)	Yes	4 (9.5)
	No	28 (66.7)
	N/A	10 (23.8)
Hep B Screening drawn in chemo unit—no. (%)	Yes	16 (38.1)
	No	16 (38.1)
	N/A	10 (23.8)
Hep B Positive—no. (%)	Yes	5 (11.9)
	NoUnknown	27 (64.3) 10 (23.8)
Infectious Disease referral in creen positive patients—no. (%)	Yes	4 (80)
	No	1 (20)
Anti-Virals prescribed in referred patients—no. (%)	Yes	1 (25)
	No	3 (75)

Abbreviations: EORLA: Eastern Ontario Regional Laboratory Association, Hep B: hepatitis B.

**Table 3:** Univariate analysis of patient and disease characteristics associated with Hepatitis B screening. * Indicates gastroesophageal compared to colorectal only. The regimen received could not be analyzed due to limited numbers outside of mFOLFOX6.

Characteristic	Description	Screened (%)	Not Screened (%)	OR (95% CI)	*p* Value
Age	Median	64	65	0.985 (0.92–1.1)	0.697
Sex	Female	16 (88.9)	2 (11.1)	3.99 (0.73–21)	0.109
	Male	16 (66.7)	8 (33.3)		
Primary Site	Appendix	2 (100)	0 (0)	0.989 (0.158–5.93)	0.945 *
	Colorectal	23 (76.7)	7 (23.3)		
	Gastroesophageal	7 (77.8)	2 (22.2)		
	Pancreatic	0 (0)	1 (100)		
Stage	II-III	13 (92.9)	1 (7.1)	6.16 (0.694–54.6)	0.103
	IV	19 (67.9)	9 (32.1)		
Intent of Therapy	Curative	14 (93.3)	1 (6.7)	6.99 (0.791–61.98)	0.0803
	Palliative	18 (66.7)	9 (33.3)		
Timing of Therapy	Neoadjuvant or Adjuvant	14 (93.3)	1 (6.7)	0.143 (0.0161–1.26)	0.0803
	First or Second Line	18 (66.7)	9 (33.3)		
Prior Systemic Therapy	Yes	11 (55)	9 (45)	17.1 (1.92–153)	0.0110
	No	21 (95.5)	1 (4.5)		
Regimen Received	mFOLFOX6	28 (80)	7 (20)		
	mFOLFOX6 + BEV	1 (33.3)	2 (66.7)		
	mFOLFOX6 + NIV	2 (66.7)	1 (33.3)		
	FOLFOXIRI	1 (100)	0 (0)		

Abbreviations: OR: odds ratio.

**Table 4:** Univariate analysis of patient and disease characteristics associated with hepatitis B positivity. * Indicates gastroesophageal compared to colorectal only.

Characteristic	Description	Hep B Positive (%)	Hep B Negative (%)	OR (95% CI)	*p* Value
Age	Median	68	63	1.04 (0.940–1.16)	0.429
Sex	Female	3 (18.8)	13 (81.3)	0.619 (0.0888–4.32)	0.628
	Male	2 (12.5)	14 (87.5)		
Primary Disease Site	Appendix	0 (0)	2 (100)	0.792 (0.0736–8.52)	0.847 *
	Colorectal	4 (17.4)	19 (82.6)		
	Gastroesophageal	1 (14.3)	6 (85.7)		
	Pancreatic	0 (0)	0 (0)		
Stage	II-III	2 (15.4)	11 (84.6)	1.03 (0.147–7.23)	0.975
	IV	3 (15.8)	16 (84.2)		
Intent of Therapy	Curative	2 (14.3)	12 (85.7)	1.20 (0.172–8.38)	0.854
	Palliative	3 (16.7)	15 (83.3)		
Timing of Therapy	Neoadjuvant or Adjuvant	2 (14.3)	12 (85.7)	0.833 (0.119–5.82)	0.854
	First or Second Line	3 (16.7)	15 (83.3)		
Prior Systemic Therapy	Yes	2 (18.2)	9 (81.8)	1.33 (0.188–9.46)	0.774
	No	3 (14.3)	18 (85.7)		

Abbreviations: Hep B: hepatitis B.

**Table 5:** Comparison of Screening Between EORLA and External Labs. * EORLA represents the laboratory at the academic cancer center. Labs included under External Lab are outside hospitals and lab companies.

	Description	EORLA *	External Lab	Total	*p* Value
Completed screening among labs ordered—no. (%)	Yes	23 (95.8)	9 (90.0)	32 (94.1)	0.508
	No	1 (4.17)	1 (10.0)	2 (5.88)	
Time to Screening Result—no. (%)	Median (Range)	3.00 (2.00–5.00)	1.00 (1.00–4.00)	3.00 (1.00–5.00)	0.121

## Data Availability

The data underlying this article will be shared at a reasonable request to the corresponding author, pending input and approval from the local REB regarding confidentiality rules.
